# Multivariate genome-wide association analysis identifies novel and relevant variants associated with anterior cruciate ligament rupture risk in the dog model

**DOI:** 10.1186/s12863-018-0626-7

**Published:** 2018-06-26

**Authors:** Lauren A. Baker, Guilherme J. M. Rosa, Zhengling Hao, Alexander Piazza, Christopher Hoffman, Emily E. Binversie, Susannah J. Sample, Peter Muir

**Affiliations:** 1 Comparative Orthopaedic Research Laboratory, School of Veterinary Medicine, University of Wisconsin-Madison, 2015 Linden Drive, Madison, Wisconsin USA; 2 Department of Animal Sciences, College of Agricultural and Life Sciences, University of Wisconsin-Madison, 1675 Observatory Drive, Madison, Wisconsin USA

**Keywords:** ACL rupture, CCL rupture, GWAS, Complex trait, Canine, Bayesian approach

## Abstract

**Background:**

Anterior cruciate ligament rupture (ACLR) is a debilitating and potentially life-changing condition in humans, as there is a high prevalence of early-onset osteoarthritis after injury. Identification of high-risk individuals before they become patients is important, as post-treatment lifetime burden of ACLR in the USA ranges from $7.6 to $17.7 billion annually. ACLR is a complex disease with multiple risk factors including genetic predisposition. Naturally occurring ACLR in the dog is an excellent model for human ACLR, as risk factors and disease characteristics in humans and dogs are similar. In a univariate genome-wide association study (GWAS) of 237 Labrador Retrievers, we identified 99 ACLR candidate loci. It is likely that additional variants remain to be identified. Joint analysis of multiple correlated phenotypes is an underutilized technique that increases statistical power, even when only one phenotype is associated with the trait. Proximal tibial morphology has been shown to affect ACLR risk in both humans and dogs. In the present study, tibial plateau angle (TPA) and relative tibial tuberosity width (rTTW) were measured on bilateral radiographs from purebred Labrador Retrievers that were recruited to our initial GWAS. We performed a multivariate genome wide association analysis of ACLR status, TPA, and rTTW.

**Results:**

Our analysis identified 3 loci with moderate evidence of association that were not previously associated with ACLR. A locus on Chr1 associated with both ACLR and rTTW is located within *ROR2,* a gene important for cartilage and bone development. A locus on Chr4 associated with both ACLR and TPA resides within *DOCK2*, a gene that has been shown to promote immune cell migration and invasion in synovitis, an important predictor of ACLR. A third locus on Chr23 associated with only ACLR is located near a long non-coding RNA (lncRNA). LncRNA’s are important for regulation of gene transcription and translation.

**Conclusions:**

These results did not overlap with our previous GWAS, which is reflective of the different methods used, and supports the need for further work. The results of the present study are highly relevant to ACLR pathogenesis, and identify potential drug targets for medical treatment.

**Electronic supplementary material:**

The online version of this article (10.1186/s12863-018-0626-7) contains supplementary material, which is available to authorized users.

## Background

Anterior cruciate ligament rupture (ACLR) is common in human beings, particularly in young, athletic individuals [1]. ACLR is a debilitating injury with a long recovery period. There is a high prevalence of early-onset knee osteoarthritis after injury [2]. Treatment of ACLR is costly; the lifetime burden of ACLR in the US is estimated at $7.6 billion annually when treated with surgical reconstruction and $17.7 billion annually if treated with rehabilitation [3]. Nearly three quarters of these cases can be classified as non-contact ruptures, typically occurring during landing or pivoting movements [4]. A clear explanation for ACLR without physical contact is not yet available, though it is generally understood that non-contact ACLR is a complex disease caused by a combination of intrinsic (variables that describe the individual) and extrinsic (variables that describe the environment of the individual) risk factors [4, 5].

Identification of intrinsic factors that increase individual risk of ACLR is an important epidemiologic goal. Thorough understanding of these risk factors will provide physicians with the means to identify individuals at high risk before they become patients in the orthopaedic surgeon’s office. Young women are up to 10× more likely to rupture their ACL compared to men [6]. Among these women, a higher than average body mass index is a significant risk factor [7, 8]. Gonadal hormones may also play a role in ACLR risk [8]. Certain characteristics of femoral and tibial morphology have also been implicated in ACLR pathophysiology. The general hypothesis underlying these risk factors is that bone morphology can alter knee joint biomechanics, potentially placing excessive stress on the ACL. For instance, it has been suggested that a narrow femoral intercondylar notch may impinge on the ACL in certain knee positions [5]. Increased posterior tibial slope (PTS) may increase anterior-directed forces on the tibia leading to greater weight-bearing load on the ACL that in certain situations may exceed its failure strength [5, 9].

Genetics plays an important and deterministic role in directing development of individual morphology and physiology. Therefore, it is perhaps unsurprising that familial analyses support the existence of genetic influence on ACLR risk. A close family history of ACLR doubles individual risk [10] and increases the risk of ACL graft rupture and contralateral ACLR [11, 12]. Candidate gene studies have reported variants in genes for collagens, proteoglycans, matrix metalloproteinases, angiogenesis-associated proteins, elastin, and fibrillin [13]. A recent genome-wide association study (GWAS) in humans did not identify any variants that met genome-wide significance but had a small case sample size of 598 individuals of various ethnic backgrounds [14]. Additionally, much of the existing research on ACLR has come from a single population, and further research is needed to confirm or deny reported associations [13, 15]. Because ACLR is a complex disease, the genetic influence on ACLR is likely composed of many variants in multiple genomic loci with small to moderate individual effects [16]. Discovery of these smaller effect loci in human populations will require a combination of very large sample sizes, often in the hundreds of thousands, high-quality phenotyping, and sophisticated statistical methods [16].

One approach to identifying genetic variants that influence complex diseases is to study the trait in a model organism that may improve GWAS utility, such as the dog. For many diseases, presenting clinical signs, pathogenesis, and treatment are extremely similar between humans and their canine counterparts [17]. The advantage of the dog lies in its unique history and genomic architecture [18]. Purebred dogs are closed populations descendent from a small number of founder individuals [18]. Selective breeding for visual and behavioral characteristics has also inadvertently selected for heritable diseases [17, 18]. This selective process has created long regions of DNA in linkage disequilibrium (LD) that likely harbor disease risk variants [18]. This combined effect allows GWAS to be performed using fewer SNP markers and smaller sample sizes than would be required to perform the same experiment in human populations. Discoveries made in the dog can then be used to inform candidate gene studies in human populations, saving great effort in the way of time and research funding.

The canine knee joint is a long-established model for human knee pathology [19]. Though there are important differences regarding weight-bearing, gait, and joint range of motion in quadrupedal mammals, studies have shown the relative dimensions of internal knee structures, including the cruciate ligaments, are similar between dogs and humans, and the canine ACL has similar cell density, blood vessel density, and cell shape compared to the human ACL [20]. ACLR is the most common cause of pelvic limb lameness in the dog [21]. Like human ACLR, the vast majority of ruptures do not involve contact injury, typically occurring while the dog is running or playing in view of the owner. As in humans, multiple risk factors have been implicated, including influence of gonadal hormones [22–24] and high body condition score [25, 26]. Anatomic risk factors that are associated with ACLR in dogs include narrow femoral intercondylar notch [27–29] distal femoral torsion [30], excessive tibial plateau angle (analogous to PTS in human beings) [31–34] and a relatively narrow tibial tuberosity width [35]. The most important risk factor for disease initiation is genetic influence, i.e. breed. While many breeds may be affected with ACLR, Newfoundlands, Rottweilers, and Labrador Retrievers are at especially high risk [23, 36]. Heritability of ACLR in dogs is moderate at 0.3-0.5 [37–39]. Variants associated with ACLR in collagen genes have been reported [40]. A GWAS in the Newfoundland breed identified variants in genes associated with neuronal signaling pathways [41]. Through linear mixed model GWA analysis, the present authors identified 128 SNPs in 99 regions that were associated with ACLR in the Labrador Retriever [39]. Gene set and pathway analysis identified enrichment for genes associated with angiogenesis, innate immune mechanisms, and extracellular matrix proteins, particularly aggrecan, which has been linked to ACLR in humans [42, 43].

Given the complex, polygenic nature of ACLR [39, 41], it is likely that additional variants remain to be identified. InPower analysis [44] of our Labrador Retriever discovery GWAS suggested that at least 172 loci influence ACLR risk in the dog [39]. While improved statistical power is most often achieved through larger sample sizes, joint analysis of multiple correlated phenotypes is an underutilized technique that has the potential to increase statistical power to detect the moderate and small effect associations expected with complex traits without the logistical concerns associated with increasing sample size [16]. To date, published GWA analyses in the dog model have all been univariate, i.e. they consider each phenotype independently. Here we present a multivariate genetic association analysis of anatomic variables, tibial plateau angle (TPA) and relative tibial tuberosity width (rTTW), with ACLR case-control status in the dog model. We identified three loci with moderate association, two of which reside in genes that have not been previously linked to ACLR but have an established role in chronic immune-mediated conditions of human beings, including rheumatoid arthritis and progression of osteoarthritis. Both of these genes have been identified as drug targets for other disorders and may be candidates for ACLR treatment.

## Methods

### Recruitment

Recruitment and quality control have been reported previously [39]. Purebred Labrador Retriever dogs were recruited through the University of Wisconsin-Madison UW Veterinary Care Hospital, online advertising, and contact with local and national breed clubs. If available, a pedigree was collected from each dog enrolled in the study to confirm purebred status. The Labrador Retriever is well-suited to GWAS because it is relatively outbred compared to some breeds with smaller population size, and has an average LD decay over distance of 20Kb [45]. This allows for a higher mapping resolution than in breeds with LD decay over distance of >1 Mb. Full siblings were excluded from the analysis to avoid bias due to over-represented genotypes in closely related individuals [46]. Control dogs were over 8 years of age [47], had stable stifles on examination, and no signs of effusion or osteophytosis on lateral radiographs [48]. Affected dogs were of any age, with examination and radiographic signs consistent with ACLR [49].

### Genotyping

Blood or saliva was collected from case and control dogs for DNA extraction. Dogs were genotyped using the Illumina Canine HD BeadChip, which contains approximately 230K single nucleotide polymorphism (SNP) markers on the CanFam3.1 reference genome. Quality control was performed using PLINK [50] and a previously established protocol [39]. Briefly, SNPs were removed for minor allele frequency <0.01, missingness >90%, and Hardy-Weinberg equilibrium of *P*<1E-7. All individuals had a genotyping call rate of >95%.

### Radiographic Knee Morphology

**Fig. 1:**
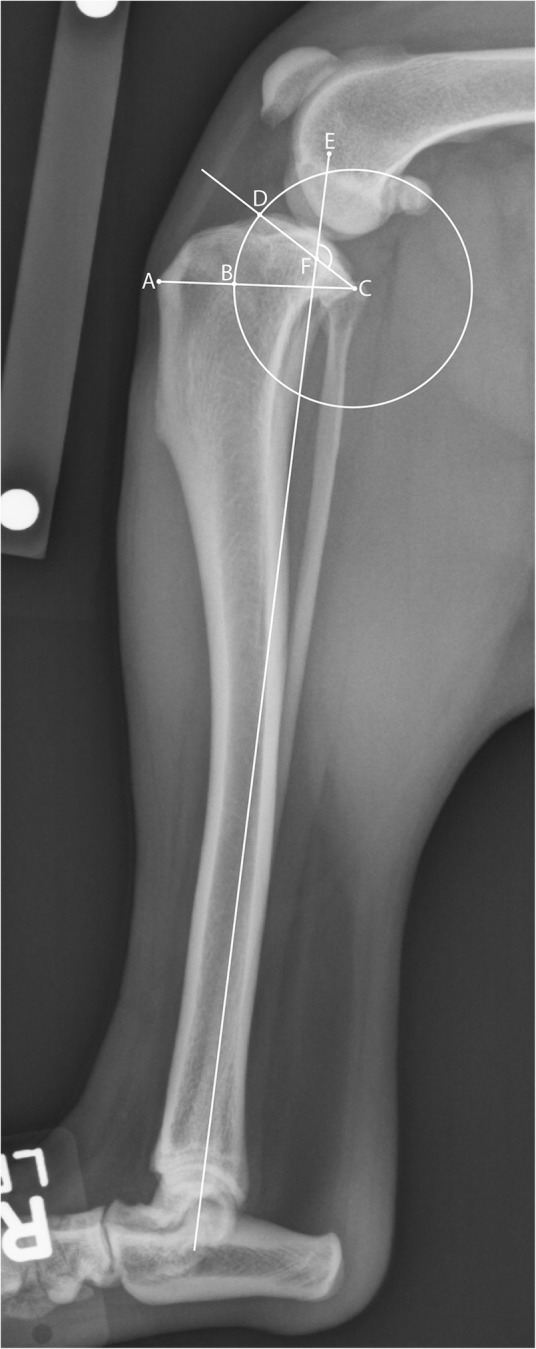
Measurements of proximal tibial morphology taken from a lateral stifle radiograph. Before measurement, the radiograph was evaluated for appropriate technique and position. A. The most anterior point of the tibial tuberosity. B. The cross point of a circle on line AC where the center is at the most posterior point of the tibial plateau (C) and also crosses the most anterior point of the tibial plateau (D). C. The most posterior point of the tibial plateau. D. The most anterior point of the tibial plateau. E. A reference line extending from the center of the intercondylar eminence proximally to the center of the tarsus distally. F. The cross point of line E and the tibial slope line (DC). Tibial plateau angle (TPA) was measured as the obtuse angle at EFC - 90°. Relative tibial tuberosity width (rTTW) was measured as the width of the tibial tuberosity divided by the width of the proximal tibia not including the tibial tuberosity (AB/BC)

Bilateral knee radiographs were evaluated to confirm that the limb was not appreciably rotated before measurement. The position of the proximal tibia relative to the proximal fibula was evaluated to assess the degree of tibial rotation along the long axis of the bone. Radiographs with substantial rotational malpositioning were removed from the analysis. TPA and rTTW were measured using Horos DICOM image viewing and measurement tools (http://www.horosproject.org). These traits are stable in skeletally mature animals. Measurements were made according to published techniques [35, 51] (Fig. 1). If bilateral radiographs were available and of adequate quality, TPA and rTTW were measured bilaterally and the final value was the average of the two measurements.

### Statistical analysis

To avoid losing dogs due to missing information, dogs with unknown weight (*n*=7 cases, 30 controls) or age (*n*=5 cases, 4 controls) were assigned the average weight or age of their case or control group before radiographic evaluation. For cases, this weight was 37.05 kilograms and age was 5.95 years. For controls, this weight was 34.16 kilograms and age was 10.53 years. Age, weight, TPA and rTTW data were tested for normality using the Shapiro-Wilk test. Measured variables were evaluated independently for differences between cases and controls using the Wilcoxon rank-sum test. Data summaries and initial statistical analyses were performed using the R statistical package [52]. To correct for population structure and confounding variables in the dataset, principal components of a genetic relationship matrix were estimated from a pruned set of SNPs with LD r^2^<0.5. Catell’s scree test was used to determine the number of principal components to retain for analysis (see Additional file 1) [53]. Case-control phenotypes were residuals of multiple logistic regression against variables known to influence ACLR in dogs [23]: age, weight, and neuter status, as well as the first 6 principal components. Before association analysis, each phenotype was quantile transformed to a standard normal distribution [54]. Genome-wide multivariate association was performed using mvBIMBAM software [54, 55]. MvBIMBAM frames association analysis as a model comparison problem whereby genotypes may be directly associated with a phenotype, indirectly associated with a phenotype (perhaps through another phenotype), or unassociated with a phenotype. The genotype may also be directly associated with more than one phenotype (referred to here as the multivariate phenotype). Bayes Factors are calculated to measure support for each model compared to the null (no association). The model comparison framework allows the user to determine not only whether an association exists between a genotype and phenotype(s), but which phenotypes are responsible for the association [55]. Bayes Factors (Log_10_ scale) were evaluated for evidence of association with the multivariate phenotype. Marginal posterior probabilities of associated SNPs were then evaluated to determine which phenotypes may be influencing the association. Regions with evidence of association were evaluated using the CanFam 3.1 Broad Improved Canine Annotation catalog [56] in the UCSC Table Browser [57].

## Results

Genotyping information was available for 336 purebred Labrador Retriever dogs. 114 dogs were excluded due to radiographs of inadequate quality, leaving 222 dogs for GWAS analysis. The final dataset contained 135,482 SNPs from 69 cases and 153 controls. The ratio of females to males in case and control groups was 0.97 and 0.88, respectively. Of the 116 males, 83 were castrated (71.5%). Of the 106 females, 87 were ovariohysterectomized (82.1%). There was no significant difference in the distribution of neutered animals across case and control groups (χ^2^=0.1583, *P*=0.69).

Before missing data imputation, average ages of case and control dogs retained for analysis were 6.4 and 10.5 years, respectively, and average weights were 35.7 and 35.3kg, respectively. A summary of statistical analysis for individual covariates and additional phenotypes is given in Table 1. Shapiro-Wilk tests confirmed all variables were not normally distributed (see Additional files 2 and 3). Therefore, Wilcoxon rank-sum tests were used to evaluate variables for differences between case and control groups. By design, dogs in the control group were significantly older than those in the case group. Dogs affected with ACLR were both significantly heavier and had a significantly smaller rTTW than unaffected dogs. Measured TPA did not differ between case and control groups.

**Table 1:** Summary statistics for individual covariates and phenotypes in ACLR case and control groups

Variables	Case	Control	*P*
Age (years)	6.4 (0.88-12.5)	10.5 (8.0-14.8)	< 2.2E-16*
Weight (kg)	37.1 (27.0 – 58.5)	34.2 (20.8-50.3)	0.00015*
TPA (degrees)	29.0 (20.9-35.0)	28.0 (20.9-37.6)	0.13
rTTW	0.64 (0.46-1.00)	0.70 (0.45-1.00)	0.00032*

All values reflect median values and range (parentheses) in case and control datasets. Values with an asterisk (*) indicate that the result was significant

*Abbreviations*: *TPA* tibial plateau angle, *rTTW* relative tibial tuberosity width

*P*-values are the result of Wilcoxon rank-sum tests performed for age, weight, TPA, and rTTW

**Fig. 2:**
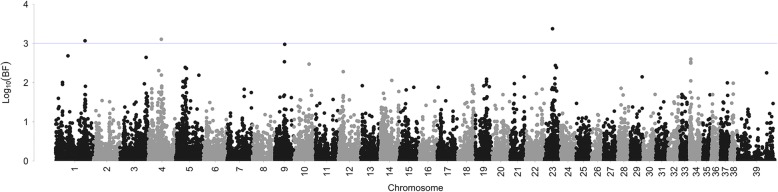
Manhattan plot of log_10_Bayes factors (BF) of the multivariate phenotype. The multivariate phenotype is the combined effect of tibial plateau angle (TPA), relative tibial tuberosity width (rTTW), and ACLR case-control status. SNPs on chromosomes 1, 4, and 23 showed moderate evidence of association with the multivariate phenotype (BF > 3)

The multivariate GWAS provided moderate evidence of association (log_10_Bayes Factor >3) for 4 SNPs with the phenotypes (Fig. 2, Table 2). Of these, two SNPs on chromosome 23 were in perfect LD and may be considered a single association. The three regions identified were on chromosomes 1, 4, and 23. The association on chromosome 1 is located within the tyrosine-protein kinase transmembrane receptor 2 gene (*ROR2*), which is involved in early formation of chondrocytes, and is required for cartilage and growth plate development. The association on chromosome 4 is within the dedicator of cytokinesis 2 gene (*DOCK2*), an important regulator of lymphocyte migration. The association on chromosome 23 is in an intergenic region close to a long non-coding RNA of unknown function**.**

**Table 2:** SNPs associated with multivariate phenotype of anterior cruciate ligament rupture (ACLR), tibial plateau angle (TPA), and relative tibial tuberosity width (rTTW)

SNP	Chr	Position	BF_mult_	BF_ACLR_	BF_TPA_	BF_rTTW_	Gene	Location	Exon Dist. (Kb)
BICF2G630788965	1	95183740	3.07	0.78	-0.23	1.75	ROR2	95124036-95282085	51.9
BICF2P1286728	4	42104780	3.10	0.68	2.18	-0.24	DOCK2	41779969-42177966	0.34
BICF2P1160758 TIGRP2P303751_rs8873754	23 23	26140588 26148612	3.37	4.24	-0.16	-0.11	(lncRNA)	26107359-26132719	N/A

Table shows log_10_Bayes Factors (BF) for multivariate association test as well as univariate tests. BF_mult_, result for multivariate phenotype; BF_ACLR_, ACL rupture only; BF_TPA_, tibial plateau angle only; BF_rTTW_, relative tibial tuberosity width only. SNPs on chromosomes 1 and 4 were located within intronic regions of genes. The distance from the SNP location to the nearest gene exon is reported (Exon Dist.). The locus on chromosome 23 resides in a region <8Kb from a long non-coding RNA (lncRNA)

An advantage of the mvBIMBAM package is the ability to discern which phenotypes in the multivariate model are responsible for the association. The posterior probability of effect (PPE) calculated for each SNP and the corresponding phenotype are displayed in Table 3. All identified SNPs showed strong probability of association with ACLR. In addition to association with the case-control phenotype, the *DOCK2* SNP was also associated with TPA and the *ROR2* SNP was also associated with rTTW. The association on chromosome 23 was primarily associated with ACLR and not TPA or rTTW. Evidence for this can be seen with higher BF_ACLR_ compared to BF_mult_ and relatively low PPE of TPA and rTTW compared to ACLR for this locus. It should be noted that PPE is the sum of the probability of a direct effect and the probability of an indirect effect on the phenotype. In all cases, the PPE was primarily explained by the probability of a direct effect on the individual phenotypes, with little influence from indirect effect.

**Table 3:** The posterior probability of effect (PPE) calculated for each SNP and corresponding phenotype

SNP	PPE_ACLR_	PPE_TPA_	PPE_rTTW_
BICF2G630788965	0.87	0.28	0.99
BICF2P1286728	0.86	0.99	0.48
BICF2P1160758 TIGRP2P303751_rs8873754	0.99	0.49	0.54

Posterior probability of effect (PPE) is 1 minus the probability of no effect on the phenotype

## Discussion

Through multivariate association analysis of ACLR case and control dogs, TPA, and rTTW, we have identified three loci with moderate evidence of association with ACLR in the Labrador Retriever model. Two of these loci are within genes that have not previously been linked to ACLR in human or dog studies, though they are highly relevant given what is known regarding ACLR pathogenesis. ACLR in dogs is a highly polygenic complex trait [39, 41], and our group previously reported 99 risk loci associated with the condition using a dataset containing many of the same dogs used in the current analysis [39]. The results of the current study should be considered complimentary and provides evidence for the benefit of using a multivariate approach to improve power to detect smaller effect associations expected when studying the genetic contribution to complex trait disease.

Before association analysis, we evaluated covariates and supplementary phenotypes for differences between cases and controls. The statistically significant difference in age between cases and controls was expected, as age was an important part of the recruitment strategy [39]. Additionally, case dogs in the sampled group weighed significantly more than controls. The association between higher body weight and increased risk of ACLR in dogs has been reported [35, 58]. While this is a notable finding, it is important to recognize that these findings do not distinguish between dogs of large body size and those that are overweight.

Several studies have evaluated the effect of excessive TPA on ACLR risk in dogs. The ACL opposes anterior tibial thrust. A steeper TPA increases anterior tibial thrust, placing increased mechanical stress on the ACL and potentially predisposing the ligament to rupture [47]. Results of case control studies of TPA on ACLR risk have been inconsistent with multiple studies declaring an excessively steep TPA as a risk factor [31–34] while others were not able to identify a significant effect [47, 59, 60]. The present study did not identify a difference in TPA between affected and unaffected dogs. However, the TPA of affected dogs was steeper than that of unaffected dogs, which is consistent with previous studies that were able to identify significant differences. Though it remains plausible that TPA has some effect on ACLR risk in dogs, it is likely that this effect is either indirect or fairly small. PTS in humans is analogous to TPA measured in dogs. Similar to dogs, human patients affected with ACLR have steeper PTS [61–63] and this effect may be more pronounced in female patients [64].

Evaluation of rTTW and ACLR status confirmed results of a previous study that reported the rTTW of dogs affected with ACLR is significantly narrower than the rTTW of unaffected dogs. Dogs with a relatively narrow tibial tuberosity experience greater anterior tibial thrust, again placing greater mechanical stress on the ACL and potentially increasing rupture risk. While our study was able to confirm rTTW as a risk factor for ACLR in dogs, a recent study in small breed dogs did not identify a difference between case and control groups [34]. It may be that risk due to rTTW is limited to large breed dogs, and this possibility should be evaluated in future epidemiological studies. To the authors’ knowledge, tibial tuberosity morphology has not been evaluated as a risk factor for ACLR in human beings.

The locus with greatest evidence of association was located on chromosome 23. This locus was primarily associated with ACLR and not TPA or rTTW. This locus lies within an intergenic haplotype block that is less than 8Kb from the transcription location of a long non-coding RNA (lncRNA) with unknown function. LncRNAs are broadly defined as long transcripts (>200 nucleotides) that do not function in a protein-coding fashion [65]. While some of these transcripts are likely non-functional, a growing list of lncRNAs have specific biological roles in many categories including cellular proliferation, chromatin remodeling, and regulation of gene transcription and translation [65, 66]. LncRNA expression tends to be highly tissue-specific [67]. The lncRNA we identified appears to be primarily expressed in testicular tissue, but not ovarian tissue [56]. Expression levels were not evaluated in tissues associated with joints (e.g. collagenous tissue or synovium). While there is evidence of gonadal influence on ACLR risk in both humans and dogs [23, 24, 68, 69], it is difficult to speculate on potential function of this lncRNA, if any, without further investigation.

The SNP association located on chromosome 4 was within the *DOCK2* gene. *DOCK2* protein interacts with Rac1 to induce lymphocyte migration into tissues, including the synovium. *DOCK2* signaling has been linked to synovitis associated with rheumatoid arthritis in human beings [70]. Lymphoplasmacytic synovitis precedes ACLR in dogs, and arthritis is typically present at the time of diagnosis [71, 72]. Additionally, numbers of lymphocytes are positively correlated with radiographic evidence of degenerative joint disease, indicating that lymphocytic inflammation likely contributes to progression of joint disease [73]. In humans, synovitis and associated lymphocytic inflammation plays a role in post-injury osteoarthritis progression and chronicity [74]. In a rabbit model of joint pathology, experimentally induced synovitis resulted in weakening of the ACL [75]. In both humans and dogs, surgical correction of joint instability fails to prevent osteoarthritis development and progression, indicating that disease progression has a substantial biochemical component [76–78]. It is feasible that aberrant *DOCK2* signaling may contribute to the inflammatory cascade associated with ACLR and play a role in both onset and progression of disease.

The SNP association located on chromosome 1 was within the *ROR2* gene, a protein with multiple roles, any of which may be relevant to ACLR. *ROR2* has a well-established role in bone development, where it is essential for appropriate patterning and chondrocyte expansion during growth [79]. *ROR2* also plays complimentary roles in both osteoblastogenesis and osteoclastogenesis from mesenchymal stem cells (MSCs) [80, 81]. Aberrant *ROR2* signaling appears to play a role in bone loss associated with rheumatoid arthritis [82] and has been shown to be significantly up-regulated in these patients [83]. *ROR2* has a more general role in cellular proliferation and migration, and this role has been implicated in several cancers of various tissue types [84, 85]. Its role in cellular expansion also has important implications for joint healing and homeostasis [79] as tissue repair requires proliferation and migration to sites of healing. Down-regulation of *ROR2* inhibits the regenerative capacity of chondrocytes [79]. Indeed, *ROR2* is down-regulated in human patients with end-stage osteoarthritis [80]. These roles indicate that altered *ROR2* signaling during development may affect the patterning and growth of long bones in a way that augments ACLR risk. Later in life, aberrant *ROR2* signaling may influence processes important for healing damaged ligament or cartilage and could contribute to post-rupture progression of osteoarthritis.

The results of the current study did not overlap with those from our previous GWAS of ACL rupture in Labrador Retrievers [39]. The present analysis was substantially different from our previous GWAS in a number of ways, and this is likely to have impacted the results. The current dataset represents dogs that had been in the previous GWAS as well as dogs that have since been added to our dataset. Therefore, while dog breed, geographical location, and recruitment strategy remained constant, the sample analyzed was substantially different from the previous analysis. Additionally, we made further correction for environmental risk factors by regressing the case-control phenotype against age, weight, and neutered status, whereas the previous study only included neutered status as a covariate. It should also be noted that the present multivariate analysis was performed using an algorithm that employs a Bayesian approach, while the previous GWAS was based on traditional linear mixed model analysis of a single case-control phenotype. It is not clear at this point how much additional variance may be explained by the variants discovered in the present study. Accurate calculation of SNP heritability (*h*^*2*^_SNP_) and heritability explained by a subset of genome-wide significant SNPs in a relatively inbred species with known relatedness is potentially complex, and will certainly require an independent sample at least as large or larger than the dataset used for this analysis [86]. Ultimately, the difference in results between the two studies highlights the need for validation in a new and larger population, as well as through other methods beyond GWAS including whole genome sequencing and gene expression studies.

## Conclusions

A multivariate analysis of ACLR, rTTW, and TPA has identified three novel variants with moderate probability of effect on ACLR. Two of these variants reside within genes that have previously been implicated in other joint pathologies, particularly rheumatoid arthritis and progression of osteoarthritis. This analysis complements our previous discovery GWAS of ACLR in the dog model, and provides further support for ACLR as a complex, multifactorial disease that may be influenced by aberrant signaling in the immune system and/or developmental variables. While multiple GWAS for ACLR have been performed in the dog model, this study is the first to identify genes directly related to joint morphology and homeostasis, which speaks to the power of the multivariate approach. Both *DOCK2* and *ROR2* represent potential drug targets for therapies targeting the biochemical aspect of ACLR and associated osteoarthritis. CPYPP is a small molecule inhibitor of *DOCK2* that may provide the scaffold for a *DOCK2-*targeting immunosuppressant [87]. As an identified oncogene, *ROR2* has been described as a candidate for targeted therapy for several cancers, and the search for an effective *ROR2* inhibitor is currently underway. Future work to validate these loci as bona fide risk factors for ACLR will provide improved understanding of ACLR pathogenesis as well as the opportunity for medical intervention through targeted drug therapy. Such advances will have an important impact on both human and animal orthopaedic health.

## Additional files

Additional file 1: Scree plot of variance explained by each principal component. The first 6 principal components were used to account for the majority of variance in the dataset. (TIFF 87 kb)

Additional file 2: Distribution of tibial plateau angle (TPA) measurements among cases and controls. (TIFF 10280 kb)

Additional file 3: Distribution of relative tibial tuberosity width (rTTW) among cases and controls. (TIFF 10280 kb)

## Abbreviations

ACLR: Anterior Cruciate Ligament Rupture
GWAS: Genome-wide association study
TPA: Tibial plateau angle
rTTW: Relative tibial tuberosity width
*ROR2*: Tyrosine-protein kinase transmembrane receptor 2 gene
*DOCK2*: Dedicator of cytokinesis 2 gene
lncRNA: Long non-coding RNA
PTS: Posterior tibial slope
LD: Linkage disequilibrium
SNP: Single nucleotide polymorphism
BF: Bayes Factor
PPE: Posterior probability of effect
MSCs: Mesenchymal stem cells
