# Supplementary figures and images for: Multivariate genome-wide association analysis identifies novel and relevant variants associated with anterior cruciate ligament rupture risk in the dog model
Source: BMC Genet. 2018 Jun 26;19:39. doi: 10.1186/s12863-018-0626-7 (PMC6019516; doi:10.1186/s12863-018-0626-7)

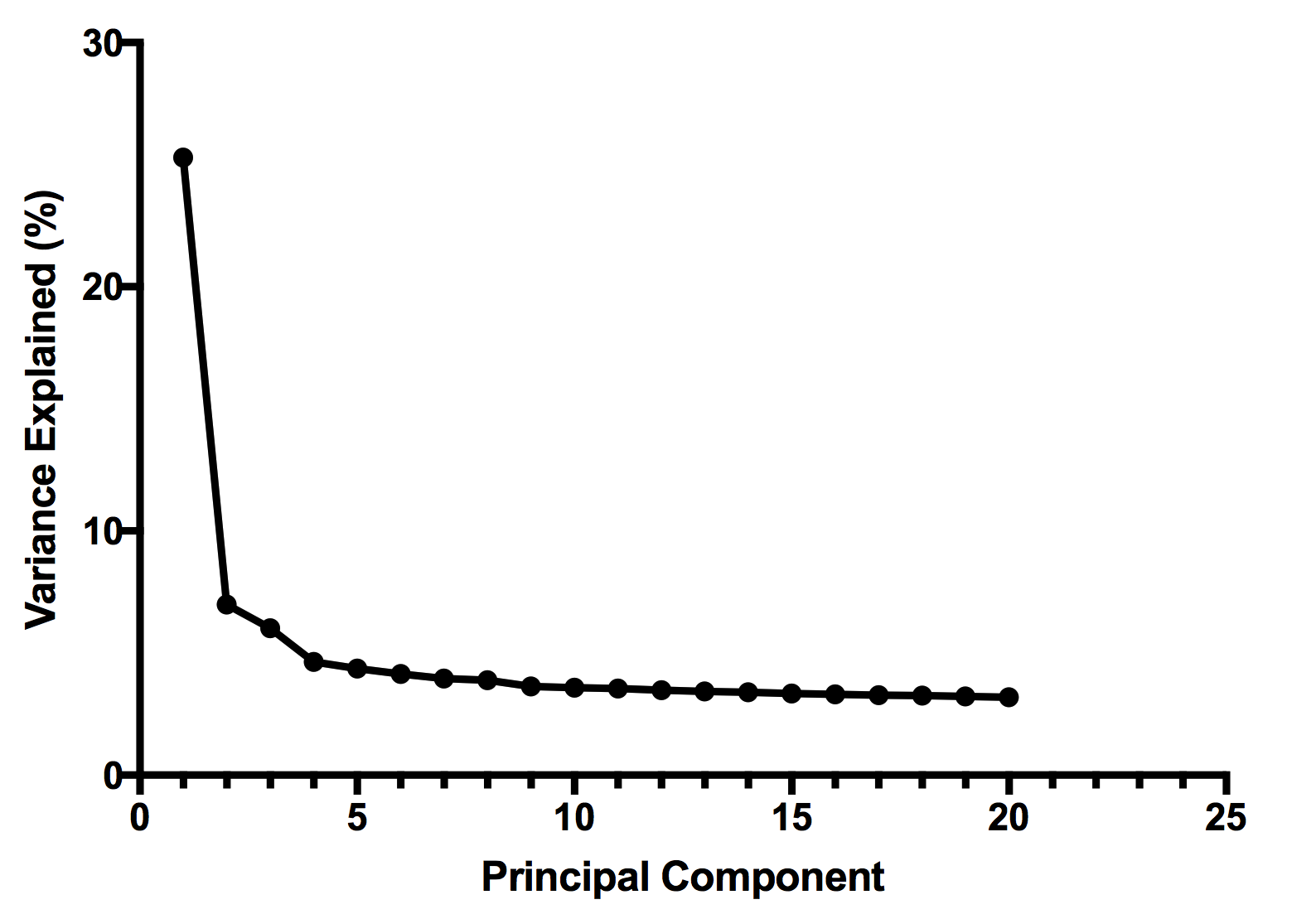

Supplement: Supplementary file 1 — Scree plot of variance explained by each principal component. The first 6 principal components were used to account for the majority of variance in the dataset. (TIFF 87 kb) [file 12863_2018_626_MOESM1_ESM.tiff]

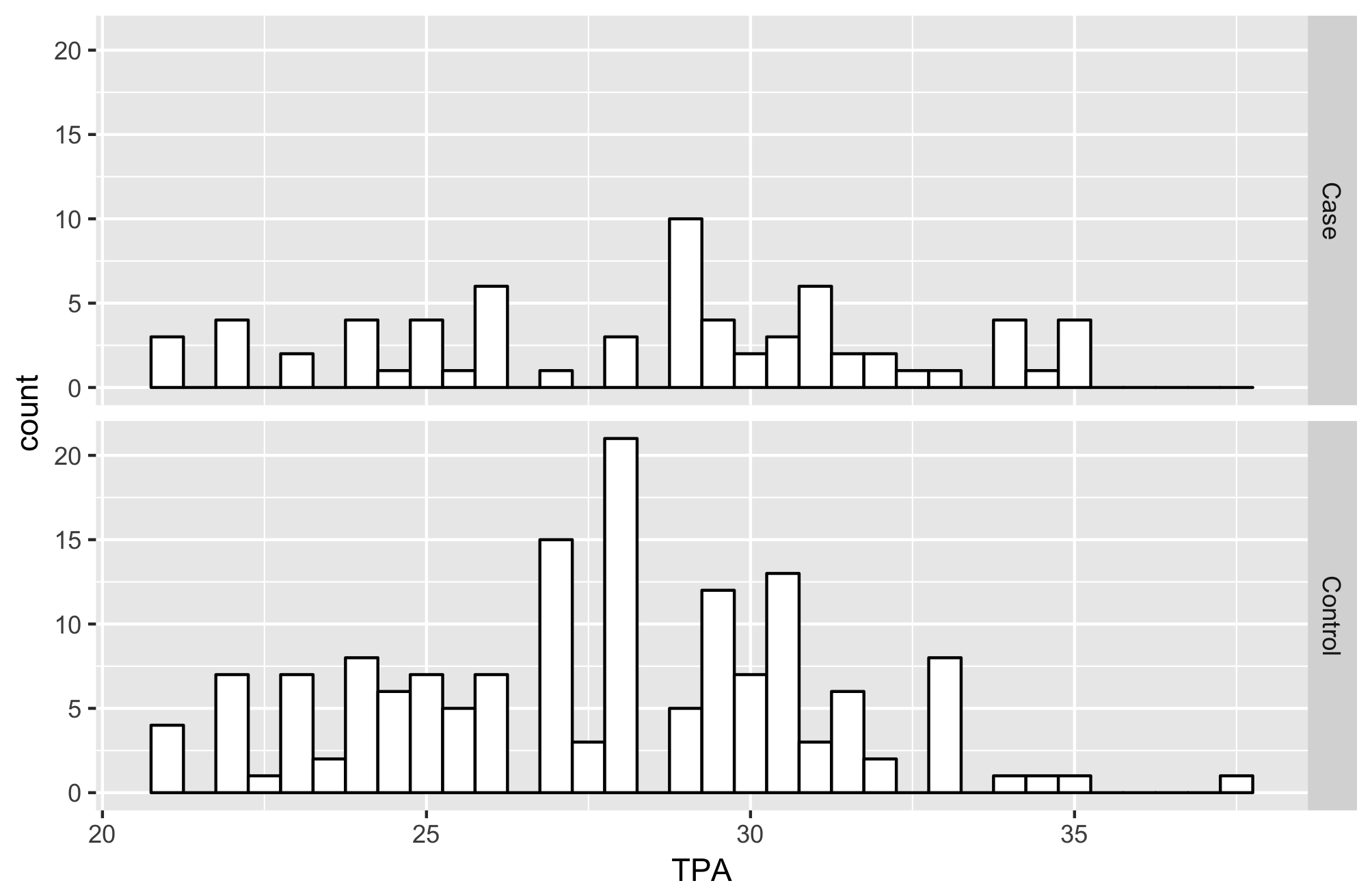

Supplement: Supplementary file 2 — Distribution of tibial plateau angle (TPA) measurements among cases and controls. (TIFF 10280 kb) [file 12863_2018_626_MOESM2_ESM.tiff]

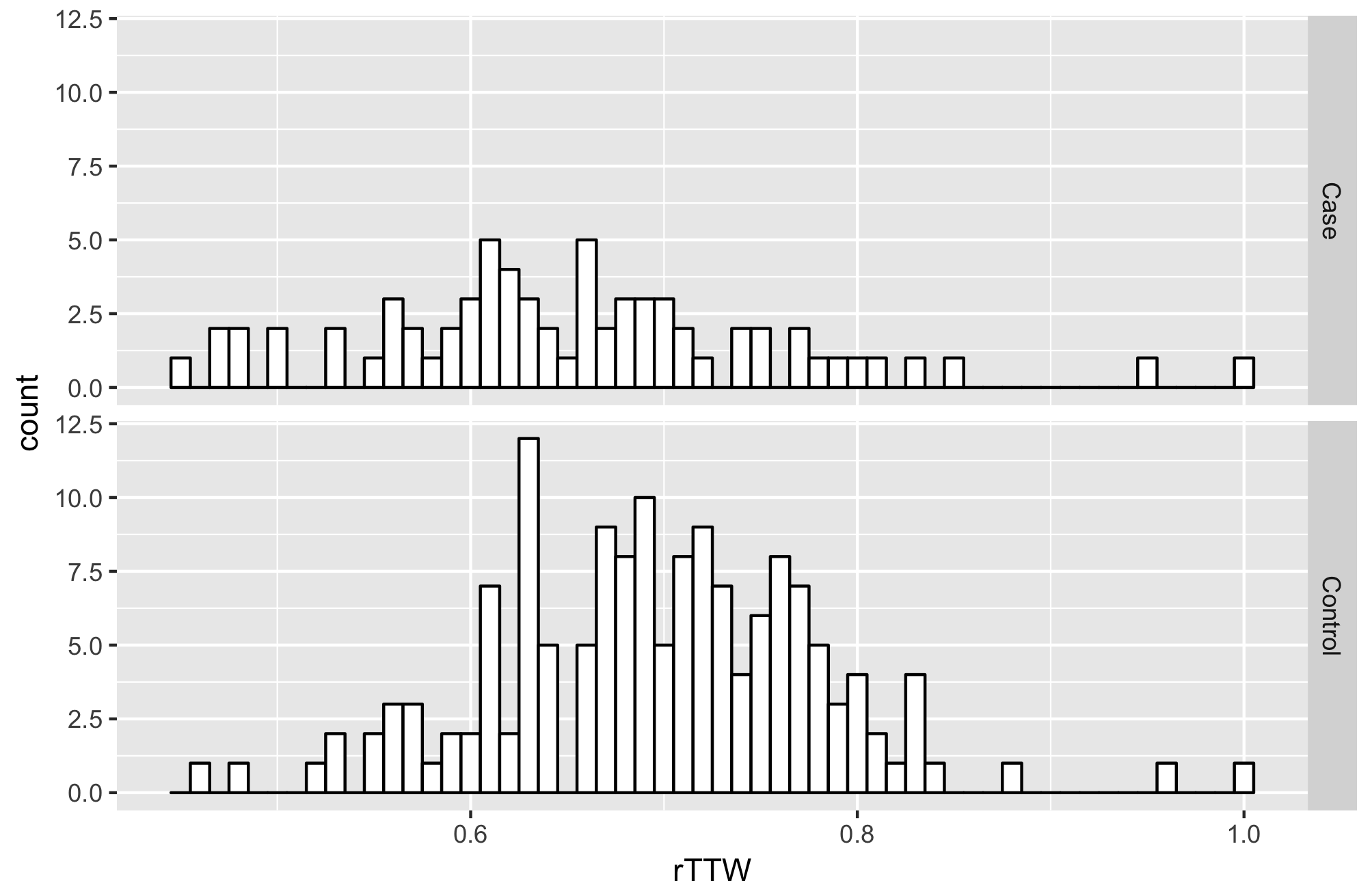

Supplement: Supplementary file 3 — Distribution of relative tibial tuberosity width (rTTW) among cases and controls. (TIFF 10280 kb) [file 12863_2018_626_MOESM3_ESM.tiff]
